# Radiotherapy treatment scheduling considering time window preferences

**DOI:** 10.1007/s10729-020-09510-8

**Published:** 2020-06-27

**Authors:** Bruno Vieira, Derya Demirtas, Jeroen B. van de Kamer, Erwin W. Hans, Louis-Martin Rousseau, Nadia Lahrichi, Wim H. van Harten

**Affiliations:** 1 Department of Radiation Oncology, Netherlands Cancer Institute - Antoni van Leeuwenhoek Hospital, Plesmanlaan 121, 1066 CX Amsterdam, The Netherlands; 2 Center for Healthcare Operations Improvement and Research (CHOIR), University of Twente, Enschede, The Netherlands; 3 Department of Industrial Engineering and Business Information Systems, Faculty of Behavioural Management and Social Sciences, University of Twente, PO Box 217, 7500 AE Enschede, The Netherlands; 4 Mathematics and Industrial Engineering, Polytechnique Montreal, 2900 Edouard Montpetit Blvd, Montreal, Quebec H3T 1J4 Canada; 5 Division of Psychosocial Research and Epidemiology, Netherlands Cancer Institute - Antoni van Leeuwenhoek Hospital, Plesmanlaan 121, 1066 CX Amsterdam, The Netherlands; 6 Rijnstate General Hospital, Arnhem, The Netherlands

**Keywords:** Mathematical programming, Radiotherapy scheduling, Patient preferences, Time windows, Operations research, Operations management

## Abstract

External-beam radiotherapy treatments are delivered by a linear accelerator (linac) in a series of high-energy radiation sessions over multiple days. With the increase in the incidence of cancer and the use of radiotherapy (RT), the problem of automatically scheduling RT sessions while satisfying patient preferences regarding the time of their appointments becomes increasingly relevant. While most literature focuses on timeliness of treatments, several Dutch RT centers have expressed their need to include patient preferences when scheduling appointments for irradiation sessions. In this study, we propose a mixed-integer linear programming (MILP) model that solves the problem of scheduling and sequencing RT sessions considering time window preferences given by patients. The MILP model alone is able to solve the problem to optimality, scheduling all sessions within the desired window, in reasonable time for small size instances up to 66 patients and 2 linacs per week. For larger centers, we propose a heuristic method that pre-assigns patients to linacs to decompose the problem in subproblems (clusters of linacs) before using the MILP model to solve the subproblems to optimality in a sequential manner. We test our methodology using real-world data from a large Dutch RT center (8 linacs). Results show that, combining the heuristic with the MILP model, the problem can be solved in reasonable computation time with as few as 2.8% of the sessions being scheduled outside the desired time window.

## Highlights

- This is the first study proposing a mathematical model for radiotherapy treatment scheduling considering patients’ preferences regarding their (daily) treatment times
- Our model optimally schedules all radiotherapy treatment sessions in less than 10 min for centers with up to 2 linacs and a one-week planning horizon
- For larger centers (3 or more linacs), we combine the model with a heuristic procedure that pre-assigns patients to linacs while maintaining a balanced workload between linacs
- The combined approach solves the problem for a large radiotherapy center with 8 linacs in less than 3.5 h of computation time with as few as 2.8% of the sessions being scheduled outside the desired 90-min time window
- The mathematical model maximizes the fulfillment of patient preferences while ensuring that all timeliness, medical and technical constraints are satisfied

## Introduction

With the increasing demand for radiotherapy (RT) services [1], which is expected to grow by an average of 16% until 2025 [2], the complexity related to the administration of existing RT resources (machines and staff) has become increasingly relevant [3, 4]. Radiotherapy treatments, usually given in a set of (daily) irradiation sessions, are administered by a machine called linear accelerator (linac), which is able to kill cancer cells by delivering high-energy radiation directed to the tumor. The growing number of treatment sessions to be booked amongst the available machines makes the scheduling process especially complex for RT centers aiming at delivering timely and patient-friendly treatments. Not only has it been shown that delays in the start of treatment may induce greater psychological distress in patients subject to longer waiting times [5], but also that 80% of the patients prefer a short interval (2 weeks or less) between referral and first oncology consultation [6]. The problem of scheduling RT treatment sessions for large varieties of treatment care pathways and technical constraints has been tackled by several studies in the current literature [7]. Models exist for assigning patients’ irradiation sessions to linacs and days [8, 9], with some studies addressing not only the scheduling component but also the sequencing of patients throughout the day [10]. While an overview on RT capacity in European countries [11] has shown that RT centers in most Western European countries are provided with enough capacity to treat all patients in due time, a survey amongst 6 Dutch RT centers within this project has shown the need to include patient preferences in the scheduling process. For these centers, asking patient preferences and integrating them into the schedule production process was common practice as they wanted to provide a better treatment experience to patients who want to maintain their routines and daily schedules during treatment. They showed that the quality of care, from a patients’ perspective, increased when patients feel involved into the scheduling process and experience the provider trying to satisfy their personal preferences for the (several) number of visits they must pay to the hospital. Moreover, literature shows that patients have different preferences regarding the time of their appointments, emphasizing the importance of fulfilling those for increased patient-centeredness [6]. The goal of these RT centers is to schedule irradiation sessions such that all patients start treatment in due time, medical and technological constraints are satisfied, and the fulfilment of patient preferences regarding the starting time of their sessions is maximized. According to these RT centers, patients have shown the desire for specific appointment times for a variety of reasons, such as avoiding traffic peak times, managing to keep their normal work schedule, or coordinating the RT treatment with their daily routines and hobbies. However, manual endeavours to produce such a schedule by (several) staff members are usually time consuming, prone to errors, and likely to find sub-optimal solutions regarding the fulfillment of patient preference requests.

Previous studies have approached different variants of the RT treatment scheduling problem and several methods have been proposed to solve it [7]. Sauré et al. [8] formulated the problem as a discounted infinite-horizon Markov decision process, showing that the percentage of treatments initiating treatment within 10 days can potentially increase from 73% to 96%. Legrain et al. [16] proposed a two-step stochastic algorithm for online scheduling of RT sessions, with results showing an average decrease in the number of patients breaching the standards of 50% for acute patients and 81% for subacute patients. Conforti et al. [9] developed an integer linear optimization program modeled in a non-block scheduling strategy, ensuring a linacs’ utilization rate of 95% while minimizing the mean waiting times. Petrovic et al. [17] propose three genetic algorithms (GAs) for minimizing waiting time target breaches when scheduling emergency, palliative and radical patients. Results showed a potential reduction of average waiting times for radical (35 to 21.48 days) and palliative (15 to 13.10 days) patients. Although efficient methods for scheduling RT sessions have been proposed, the literature in relation to optimizing the sequencing of patients throughout the day is rather scarce [7]. However, as discussed above, besides complying with timeliness requirements and technical constraints, RT centers are often faced with the problem of finding a schedule that maximizes patient preferences regarding the starting time of irradiation sessions. In more recent years, two models have been proposed [12, 15] to optimize intra-day linac schedules in a way that starting time of irradiation sessions do not deviate from a pre-defined target time by more than a certain threshold (30 min in both [12] and [15]). Vogl et al. [15] modeled the problem for an ion beam facility (in which a single particle beam serves multiple treatment rooms). They included time window constraints whose violations are penalized in the objective function, which minimizes the idle time of the particle beam unit. Using real-world inspired data, they found that a combination of two stand-alone metaheuristic approaches leads to the best results when compared to a genetic algorithm and iterated local search. Maschler and Raidl [12], on the other hand, proposed an enhanced iterated greedy (EIG) metaheuristic to solve the patient scheduling problem with limited starting time variation between sessions in particle therapy. Computational experiments using fictitious data showed that the EIG method outperforms two other metaheuristics in 26 out of 30 instances. However, these two studies focus on particle therapy (PT), and thus cannot be applied directly to conventional external-beam RT since the technical and medical constraints vary considerably. For instance, in particle therapy a single beam source is used by multiple treatment rooms, but only one room can use the beam source at a time. Moreover, in both studies, patient preferences are not taken into account, and only approximation methods are able to solve the problem in acceptable time due to the complexity of the mathematical formulations and high number of constraints involved. On a hospital-wide setting, Gartner et al. [13] present exact and heuristic methods for the scheduling and routing of physical therapists where scheduled treatment sessions are bounded to pre-defined time windows. However, they optimize their models for the minimization of waiting times only and do not allow for sessions to be scheduled outside the required time window.

Overall, most models presented in the current literature focus on deciding on the specific day and linac of each irradiation session, with the sequencing of patients in each linac and each day being either neglected or determined in a secondary stage. Studies addressing the sequencing problem considering time windows are developed in the context of PT, thus they are not directly applicable to conventional RT. No studies have been found where optimization models integrate patient preference structures when deciding on the appointment times of irradiation sessions in conventional external-beam RT. In this paper, we propose a mixed-integer linear programming (MILP)-based approach for scheduling and sequencing RT treatment sessions. Our model takes all the medical and technical constraints into account, and maximizes the satisfaction of time window preferences given by patients for the starting time of their appointments. To solve the problem more efficiently for larger instances, we propose a heuristic procedure that pre-assigns patients to linacs before using the MILP model to solve each of the subproblems (subset of patients and linacs) independently. We compare the performance of the MILP model alone and the combined approach regarding solution quality and CPU time for different instance sizes. The feasibility of our algorithm is tested using real-world data from the Netherlands Cancer Institute (NKI), a large RT center located in Amsterdam, the Netherlands, with approximately 5000 new treatments per year and 8 linacs operating on a daily basis. Although patient preferences are currently not recorded by the NKI and thus data regarding patient preferences is not available, we have performed a sensitivity analysis on both the preference structure breakdown and the size of the possible time windows being made available for patients to chose from.

This paper is organized as follows: Section 2 contains the formal problem description. The methodology including our MILP model and the algorithm to pre-assign patients to linacs are presented in Section 3. Section 4 presents the computational experiments performed using real-world data from a large RT department. The analysis and discussion of the results are described in Section 5, and Section 6 outlines the major conclusions of this study.

## Problem description

In the RT scheduling problem, the aim is to schedule a set of treatment sessions for a set of cancer patients $\mathcal {P}$ over a given planning horizon $\mathcal {T}$, discretized in time periods $t =1, ... , |\mathcal {T}|$. Each patient has a certain due date *d*_*i*_, which defines the maximum date a patient should start treatment before the maximum waiting time target is achieved. Treatment sessions are delivered by a set of linear accelerators $\mathcal {K}$. The capacity of each linac is given by the number of available time slots $\mathcal {|S|}$ of duration *l*. Each session of each patient $i \in \mathcal {P}$ has an estimated processing duration quantified as a pre-defined number of time slots *p*_*i*_. Most sessions are delivered on a daily basis, however some patients (e.g. hypofractionation schemes) may require (at least) one day off between two consecutive treatment sessions. Typically, every linac is capable of treating patients from all tumor types. However, some RT centers such that of the NKI may have a master schedule (pre-allocation) indicating that some patient groups must be assigned to a restricted set of linacs. For instance, brain patients may only be allowed to be scheduled on the technologically most advanced linacs as patients with this tumor site benefit the most from higher precision levels of the linac’s delivery. Factors such as the accuracy level and other technologies (such as cone-beam CT) of the linacs, departments may want pre-allocate certain patient groups to certain linacs. In case a pre-allocation exists, each patient must receive treatment in one of the set of linacs pre-allocated to his/her patient group ($\mathcal {K}^{i}$). The duration of treatment sessions typically vary per patient group, but sessions of each individual patient usually have the same duration throughout the whole treatment. Besides, we assume that RT centers aim at delivering all RT sessions on the same linac for each given patient. Although there are no technical or medical constraints that enforce this as a necessary condition, from a patient perspective it is highly desirable that patients receive their daily sessions on the same linac such that they always see the same facilities and personnel throughout most of the treatment. In addition, due to the combination of RT with other treatment modalities, such as chemotherapy, some patients may need to start treatment on a Monday to guarantee a proper coordination between the different treatment modalities. Moreover, because the first irradiation fraction of each patient may take longer than expected due to the need of explaining the whole process to the patient, RT centers commonly set a threshold *T* limiting the number of new patients who are scheduled to start treatment on the same linac and the same day in order to avoid congestion. Besides, for some patients there may be the need of guaranteeing that specialized staff (e.g. doctors) are in the department during the delivery of irradiation sessions to certain patients ($\mathcal {P}^{f}$) in case unexpected complications occur. In these cases, a time frame [$\underline f^{t},\overline f^{t}$] must be set to bound the starting time of all irradiation sessions of those patients.

**Fig. 1:**
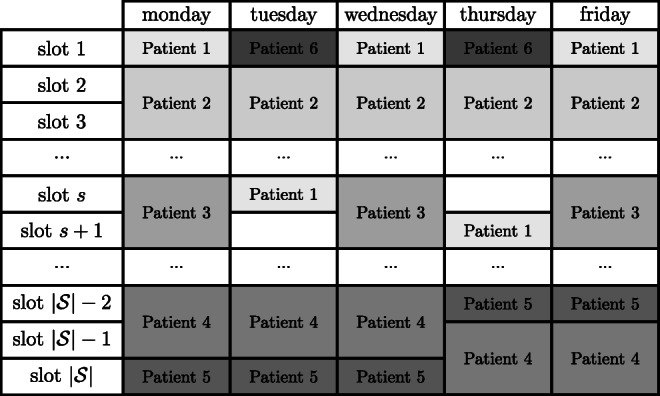
Example of a weekly schedule of a linear accelerator in RT

Apart from the fulfillment of all the medical and technological constraints, in this problem we consider that RT centers are interested in finding a (weekly) schedule that minimizes the number of appointments scheduled outside the preferential time window requested by patients. This means that RT centers can run the model during the last workday of the previous week (i.e. Friday). Thus, data regarding (regular) patients to be scheduled is known by the beginning of the planning horizon, allowing to build a deterministic model to be used at an offline operational level (for a definition of the different hierarchical planning levels, see Hulshof et al. [14]). Although other sources of uncertainty (session duration, no-shows, sessions’ cancellations, linac breakdowns) exist, we verified that the percentage of occurrences of deviations between the planned and the realized values was lower than 1% for each single case. Thus, we assumed these input parameters as deterministic, focusing on the performance of the “planned” solution regardless of the modifications that may be required at an online operational level. In our model, the goal is that the starting time of the scheduled sessions fall within the patients’ desired time window $[t_{i}^{\min \limits },t_{i}^{\max \limits }]$ consistently. Figure 1 depicts a possible weekly schedule of a linear accelerator in external-beam RT with time window preferences. Note that appointments times are, for most patients, consistent throughout the week. Let us assume that, in this schedule, Patient 1 had requested their sessions to be booked between slots 1–3 inclusive. Then, two (Tuesday and Thursday) out of five sessions would fall outside the desired window. Considering all appointments of all other patients in Fig. 1 are set within the requested time window, then the performance value of the solution for this linac would be equal to 23/25, i.e. 92% of the appointments are booked within the requested time window. In this paper, we propose a method that aims at maximizing the percentage of sessions falling within the requested time window for patients and linacs of real-world RT centers.

## Methodology

In this section we present the methodology developed to solve the RT scheduling problem with time window preferences given by patients for the starting time of their sessions. We use the notation presented in Table 1 to formulate the MILP model and the heuristic procedure designed to pre-allocate patients to linacs presented in Algorithm 1.

**Table 1:** Notation of the MILP model and heuristic procedure

Parameter	Description
$\mathcal {P}$	Set of patients to be scheduled ($i,j \in \mathcal {P}$)
$\mathcal {K}$	Set of linear accelerators ($k \in \mathcal {K}$)
$\mathcal {S}$	Set of time slots per linac ($s \in \mathcal {S}$)
$\mathcal {T}$	Set of time periods (days) in the planning horizon ($t \in \mathcal {T}$)
$\mathcal {P}^{n}$	Set of patients who have not started treatment ($\mathcal {P}^{n} \subseteq \mathcal {P}$)
$\mathcal {P}^{m}$	Set of patients who must start treatment on monday ($\mathcal {P}^{m} \subseteq \mathcal {P}$)
$\mathcal {P}^{f}$	Set of patients with restricted time frame for treatment sessions ($\mathcal {P}^{f} \subseteq \mathcal {P}$)
$\mathcal {K}^{i}$	Set of feasible linacs for treating patient *i* ($\mathcal {K}^{i} \subseteq \mathcal {K}$)
*T*	Maximum number of patients starting treatment on the same linac and same day
*l*	Time slot duration, in minutes, in each linac, each day
*a*_*kst*_	1 if slot *s* of linac *k* is available on time period *t*, 0 otherwise
$\underline f^{t}, \overline f^{t}$	Lower and upper bound of the restricted time frame set for time period *t*
*I*_*i*_	Number of total remaining sessions to be delivered to patient *i*
*d*_*i*_	Due date: time period by which patient *i* must start treatment
*p*_*i*_	Duration, in number of time slots, of each session of patient *i*
*b*_*i*_	Number of time periods needed between sessions of patient *i* (1 for consecutive daily sessions)
$t_{i}^{\min \limits },t_{i}^{\max \limits }$	Lower and upper bound of the time window preference for patient *i*
*c*_*i*_	Linac in which patient $i \notin \mathcal {P}^{n}$ is currently undergoing treatment
*C*_*k*_	Weekly capacity, in minutes, of linac *k*
*vol*(*i*)	Expected weekly session time, in minutes, for patient *i*
*WL*(*k*)	Workload, in minutes of session time, assigned to linac *k*
Variable	Description
$x_{iks}^{t}$	1 if patient *i* is scheduled a session starting on time slot *s* of linac *k* in day *t*, 0 otherwise
$y_{ik}^{t}$	1 if new patient *i* starts treatment in period *t* and linac *k*, 0 otherwise
${\Delta }^{-}_{it},{\Delta }^{+}_{it}$	Lower and upper deviation, in minutes, from preference time window of patient *i* in time period *t*

### Mathematical programming model

The problem is formulated such that the capacity of each linac is divided in time slots $s = 1, ... , |\mathcal {S}|$ of duration *l*. When scheduled, patients’ sessions are assigned a certain starting time slot on a certain linac and day. To this end, we introduce binary variables $x_{iks}^{t}$, which take the value 1 if patient *i* is scheduled for a session starting on time slot *s* of linac *k* in day *t*, and 0 otherwise. If a certain starting slot is assigned to a patient, we prevent the following slots needed to achieve the corresponding patient’s session duration on that same linac and day from being assigned to other patients. The objective (1) is to minimize the overall deviation between the bounds of the preferred time window $[t_{i}^{\min \limits },t_{i}^{\max \limits }]$ given by patients and the starting time of their appointments. Real variables ${\Delta }^{-}_{it}$ and ${\Delta }^{+}_{it}$ are used to represent this deviation for each patient in each day. Binary variables $y_{ik}^{t}$ are auxiliary variables, which will be equal to 1 if a new patient starts his/her treatment in period *t* and linac *k*, and 0 otherwise.

#### Objective function

The objective (1) is to minimize the overall deviation between the bounds of the preferred time window $[t_{i}^{\min \limits },t_{i}^{\max \limits }]$ given by patients and the starting time of their appointments. Real variables ${\Delta }^{-}_{it}$ and ${\Delta }^{+}_{it}$ are used to represent this deviation for each patient in each day. Binary variables $y_{ik}^{t}$ are auxiliary variables, which will be equal to 1 if a new patient starts his/her treatment in period *t* and linac *k*, and 0 otherwise.

1 $$ \min \sum\limits_{i \in \mathcal{P}\setminus\{1\}} \sum\limits_{t \in \mathcal{T}} ({\Delta}^{-}_{it} + {\Delta}^{+}_{it})  $$

The technical and medical constraints described in Section 2 are modeled as follows:

#### Sessions’ assignment constraints

Inequalities (2)–(4) ensure that patients receive their sessions on the same linac and with the required frequency *b*_*i*_ until the number of sessions or the end of planning horizon is reached. Constraints (2) and (3) force the necessary sessions to be booked, at least every *b*_*i*_ days, as soon as a first session is scheduled. Constraints (4) avoid unnecessary sessions from being scheduled in days occurring between the days of the sessions booked by constraints (2)–(3) when *b*_*i*_ > 1.

2 $$ \begin{array}{@{}rcl@{}} &&{}\sum\limits_{s \in \mathcal{S}} x_{iks}^{t} - \sum\limits_{s \in \mathcal{S}} \sum\limits_{t'=1}^{t-1} x_{iks}^{t^{\prime}} \leq \sum\limits_{s \in \mathcal{S}} x_{iks}^{n}, \\ &&{} \forall i \in \mathcal{P}, \forall k \in \mathcal{K}, \forall t = 2, ... , \mathcal{T}, \\ &&{} \forall n = t+b_{i},t+2b_{i}, ... , \min\{|\mathcal{T}|, t + b_{i}(I_{i} - 1)\} \end{array} $$

3 $$ \begin{array}{@{}rcl@{}} &&{}\sum\limits_{s \in \mathcal{S}} x_{iks}^{1} \leq \sum\limits_{s \in \mathcal{S}} x_{iks}^{n}, \forall i \in \mathcal{P}, \forall k \in \mathcal{K}, \\ &&{}\forall n = b_{i}+1,2b_{i}+1, ... , \min\{|\mathcal{T}|, b_{i}(I_{i} - 1)+1\} \end{array} $$

4 $$ \begin{array}{@{}rcl@{}} &&{}1 - \sum\limits_{s \in \mathcal{S}} x_{iks}^{t} \geq \sum\limits_{s \in \mathcal{S}} x_{iks}^{n}, \forall i \in \mathcal{P}, \forall k \in \mathcal{K}, \\ &&{}\forall t = 1, ... , |\mathcal{T}|-b_{i}, \forall n = t + 1, ... ,  t + b_{i} - 1, b_{i} \geq 2 \\ \end{array} $$

#### Limitations on the number of sessions

Inequalities (5) limit the number of sessions that each patient can receive to a maximum of one per day. Constraints (6) restrict the number of sessions delivered during the planning horizon to the number of remaining sessions for that patient.

5 $$ \begin{array}{@{}rcl@{}} &&{}\sum\limits_{k \in \mathcal{K}} \sum\limits_{s \in \mathcal{S}} x_{iks}^{t} \leq 1, \forall i \in \mathcal{P}, \forall t \in \mathcal{T} \end{array} $$

6 $$ \begin{array}{@{}rcl@{}} &&{}\sum\limits_{k \in \mathcal{K}} \sum\limits_{s \in \mathcal{S}} \sum\limits_{t \in \mathcal{T}} x_{iks}^{t} \leq I_{i}, \forall i \in \mathcal{P} \end{array} $$

#### Timeliness constraints

Constraints (7) impose that every patient starts treatment before their due date *d*_*i*_. Note that for patients who need to start treatment on a Monday one can set *d*_*i*_ = 1.

7 $$ \begin{array}{@{}rcl@{}} \sum\limits_{k \in \mathcal{K}} \sum\limits_{s \in S} \sum\limits_{t=1}^{d_{i}} x_{iks}^{t} \geq 1, \forall i \in \mathcal{P} \end{array} $$

#### Linacs’ capacity constraints

Constraints (8) ensure that each (available) slot of each linac is scheduled at most one session per day, and Restrictions (9) ensure that each patient is assigned to a feasible linac by preventing sessions of being assigned to slots of linacs that do not belong to $\mathcal {K}^{i}$.

8 $$ \begin{array}{@{}rcl@{}} &&{}\sum\limits_{i \in \mathcal{P}} x_{iks}^{t} \leq a_{kst}, \forall k \in \mathcal{K}, \forall s \in \mathcal{S}, \forall t \in \mathcal{T} \end{array} $$

9 $$ \begin{array}{@{}rcl@{}} &&{}\sum\limits_{s \in \mathcal{S}} \sum\limits_{t \in \mathcal{T}} x_{iks}^{t} \leq 0, \forall i \in \mathcal{P}, \forall k \in \mathcal{K}\setminus\{\mathcal{K}^{i}\} \end{array} $$

#### Maximum number of patients starting treatment per linac per day

Constraints (10)–(11) force variables $y_{ik}^{t}$ to take the value 1 if a new patient *i* starts treatment on linac *k* and day *t*, while Eq. 12 use these auxiliary variables to limit the number of patients starting treatment on the same linac and same day to the pre-defined threshold *C*.

10 $$ \begin{array}{@{}rcl@{}} &&{} y_{ik}^{t} \geq \sum\limits_{s \in \mathcal{S}} x_{iks}^{t} - \sum\limits_{s \in \mathcal{S}} x_{iks}^{t^{\prime}}, \forall i \in \mathcal{P}^{n}, \forall k \in \mathcal{K}, \forall t = 2, ... ,  \mathcal{T}, \\ &&{} t^{\prime} = \max\{1,t-b_{i}\} \end{array} $$

11 $$ \begin{array}{@{}rcl@{}} &&{} y_{ik}^{1} \geq \sum\limits_{s \in \mathcal{S}} x_{iks}^{1}, \forall i \in \mathcal{P}^{n}, \forall k \in \mathcal{K} \end{array} $$

12 $$ \begin{array}{@{}rcl@{}} &&{} \sum\limits_{i \in \mathcal{P}^{n}} y_{ik}^{t} \leq C, \forall k \in \mathcal{K}, \forall t \in \mathcal{T} \end{array} $$

#### Session duration constraints

Restrictions (13) prevent the remainder of the time slots needed to achieve the session duration *p*_*i*_ after the chosen starting slot ($x_{iks}^{t}$) from being assigned to other patients on the same linac and day. Inequalities (14) ensure that the starting slot of sessions with a duration of two or more slots are not assign to the last slot(s) of the day.

13 $$ \begin{array}{@{}rcl@{}} &&{}x_{iks}^{t} \leq 1 - \sum\limits_{i^{\prime} \in \mathcal{P}} x_{i^{\prime},k,s^{\prime}}^{t}, \forall i \in \mathcal{P}, \forall k \in \mathcal{K}, \\ && {}\forall s=1, ... , |\mathcal{S}|-p_{i}+1, \forall t \in \mathcal{T}, \\ &&{}\forall s^{\prime}=s+1, ... , s+p_{i}-1, p_{i} \geq 2 \end{array} $$

14 $$ \begin{array}{@{}rcl@{}} &&{}x_{iks}^{t} = 0, \forall i \in \mathcal{P}, \forall k \in \mathcal{K}, \forall s=|\mathcal{S}|-p_{i}+2, ... , S, \\ &&{}\forall t \in \mathcal{T}, p_{i} \geq 2 \end{array} $$

#### Time window constraints

Constraints (15) force treatment sessions of each patient to fall within the restricted time frame set by the department due to the need of ensuring that specialized staff are present during the sessions of the applicable patients ($\mathcal {P}^{f}$). Equation 16 set variables ${\Delta }^{-}_{it}$ and ${\Delta }^{+}_{it}$ to take a non-zero value if a session’s starting time deviates from the desired lower and upper bounds, respectively, and constraints (17) are the non-negativity constraints associated with the real variables.

15 $$ \begin{array}{@{}rcl@{}} && {}x_{iks}^{t} \leq 0, \forall i \in \mathcal{P}^{f}, \forall k \in \mathcal{K}, \forall s \in \mathcal{S}, \forall t \in \mathcal{T}, \\ &&{}s < \underline f^{t}, s > \overline f^{t} \end{array} $$

16 $$ \begin{array}{@{}rcl@{}} &&{}t_{i}^{\min}x_{iks}^{t} - {\Delta}^{-}_{it} \leq l(s-1)x_{iks}^{t} \leq t_{i}^{\max}x_{iks}^{t} + {\Delta}^{+}_{it}, \\ &&{}\forall i \in \mathcal{P}, \forall k \in \mathcal{K}, \forall s \in \mathcal{S}, \forall t \in \mathcal{T} \end{array} $$

#### Non-negativity and integrality constraints

17 $$ \begin{array}{@{}rcl@{}} &&{}{\Delta}^{-}_{it} \geq 0, {\Delta}^{+}_{it} \geq 0, \forall i \in \mathcal{P}, \forall t \in \mathcal{T} \end{array} $$

18 $$ \begin{array}{@{}rcl@{}} &&{}x_{iks}^{t},y_{ik}^{t} \in \mathbb{B}, \forall i \in \mathcal{P}, \forall k \in \mathcal{K}, \forall s \in \mathcal{S}, \forall t \in \mathcal{T} \end{array} $$

### Patient-to-linac assignment

As we demonstrate in Section 4, the proposed MILP model alone is not capable of solving the problem for larger RT centers (5 linacs or more) in acceptable computation time. In these cases, we apply a heuristic procedure (Algorithm 1) to pre-assign patients to linacs, and use the MILP model to solve the sequencing problem for each subset of linacs, hereby referred to as “clusters”. In Algorithm 1, *C*_*k*_ represents the weekly capacity of the linacs, in minutes, *vol*(*i*) represents the total session time expected during the whole planning horizon for patient *i*, while *WL*(*k*) contains the workload, measured in total minutes of session time, in each linac *k*. 
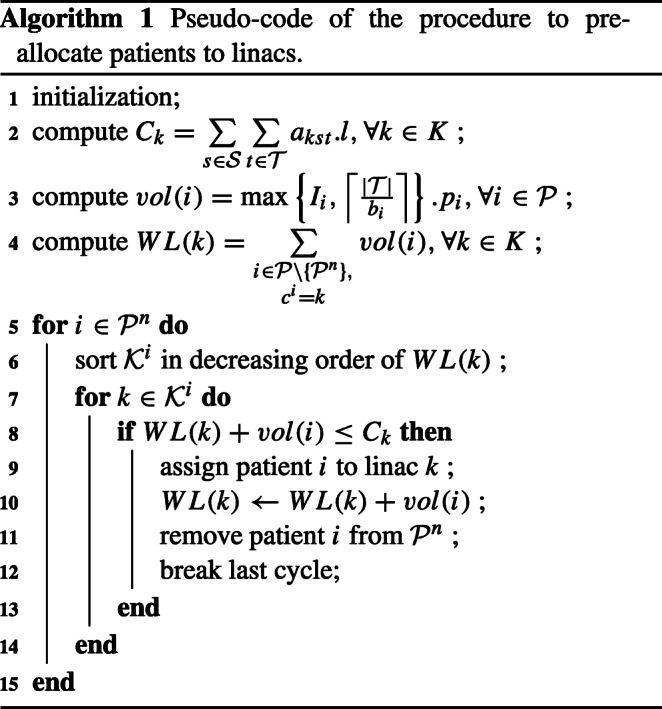


The algorithm initiates by computing the initial values of each parameter. Note that patients undergoing treatment ($i \in \mathcal {P}\setminus \mathcal \{\mathcal {P}^{n}\}$) are already assigned to a linac, and thus the initial values *WL*(*k*) will be given by the total patient volume undergoing treatment on linac *k* by the beginning of the planning horizon. The variable *WL*(*k*) associated with linac *k* keeps track of the total volume (number of sessions times the duration) assigned to that linac. Next, and for each new patient $i \in \mathcal {P}^{n}$, the algorithm sorts the set feasible linacs $\mathcal {K}^{i}$ in increasing order of *WL*(*k*). This way, the algorithm first searches the less busy linacs to foster a balanced workload. Starting from the top of the list of feasible linacs for patient *i*, the algorithm checks whether the current patient’s volume *vol*(*i*) fits the current available capacity *C*_*k*_ and, if so, assigns the patient to that linac, updating the value of *WL*(*k*) by the total volume *vol*(*i*) of the patient being assigned. If the patient has been assigned to *k*, patient *i* is removed from the list and the algorithm proceeds to the next patient. If not, the procedure continues to search for the next linac on the list until a feasible linac is chosen. Algorithm 1 therefore assumes that there is enough linac capacity to treat the whole patient population $\mathcal {P}$ being scheduled in order to find a feasible pre-assignment solution.

## Computational experiments

This section presents the results of the computational experiments we have performed with our model. Section 4.1 describes the instance generator using historical patient data from the RT department of the NKI. Section 4.2 shows the results for several instance sizes using the NKI historical records to generate patient data for a reduced number of linacs. In Section 4.3 we solve the problem for the NKI size (8 linacs) using a method that combines our MILP model and the Algorithm 1, and Section 4.4 describes a sensitivity analysis performed to analyze the impact of the variation of patient requests and the competition for the same time window.

The MILP model and Algorithm 1 were coded in C++ using Visual Studio 2017 and the Concert Technology of CPLEX v12.8.0, which was used as a solver. All experiments were conducted on a desktop computer with a processor Intel i7 3.6 GHz and 16 GB of RAM using up to 8 threads, running on a 64-bit version of Windows 10. In our case study, the goal is to find a weekly schedule (Monday to Friday), which means that RT centers can run the model during the last workday of the previous week (i.e. Friday) so that the maximum amount of patient data is known. The maximum allowed CPU time was set to 28800 s (8 h) per run.

### Historical patient data used for generating test instances

Patient characteristics are generated according to empirical distributions generated using historical data collected throughout 2017 (number of new treatment courses = 4720). In our instance generator, we start by randomly attributing a care plan (i.e. care trajectory) to each patient. There are 56 care plans in total, with the largest being “Bone metastasis” (23.3%) and “Breast” (16.5%). Thereafter, we generate the number of sessions *I*_*i*_ of each patient, which can vary between 1 and 35 sessions depending, to a large extent, on the care plan. For instance, nearly half of all prostate patients will undergo 35 sessions, while 65% of all bone metastasis patients are prescribed 3 sessions or less. Similarly, the urgency level of each patient, which can be either urgent (34%) or regular (66%), is randomly assigned according to historical data associated with his/her care plan. The due date (*d*_*i*_ = 1, ... , 5) of new patients (35% of the patient population) is generated as follows: we calculate, for each patient, the difference between the maximum waiting time according to the standards defined by the Dutch Society for Radiation Oncology (NVRO) [18] (21 days for urgent patients, and 28 days for regular patients) and the number of days elapsed from referral to treatment planning. For instance, if for a regular patient *i*, who needs to start treatment within 28 days of referral, the pre-treatment phase (referral to treatment planning) took 25 days to complete, this value would be equal to 3. We use the values verified in practice and corresponding proportions in the whole 2017, per care plan, to build empirical distributions from which due dates *d*_*i*_ are randomly generated. The due date will be equal to 1 in case a patient is already undergoing treatment, and in case of new patients who need to start their treatment on a Monday ($\mathcal {P}^{m}$). The target due date already takes into account eventual delays that may be required due to medical or personal reasons, which are usually known by the beginning of the pre-treatment phase (consultation) and will be reflected in the waiting time target date set by the department. Furthermore, the duration of each session *p*_*i*_ is also assigned empirically on a care plan basis, ranging from 10 to 30 min, in multiples of 5 min. Data shows that the majority of patients will be scheduled 15-min sessions (60.5%), with 19.9% patients having sessions of 20 min or longer.

The daily available time for delivering irradiation sessions in the clinic ranges from 07h30 to 17h30, thus $|\mathcal {S}| = 120$ by considering *l* = 5 min. We solve the problem for a planning horizon of one labour week, discretized in time periods of one day ($|\mathcal {T}|=5$). The restricted time frame ($\underline w^{t}, \overline w^{t}$) due to the need of guaranteeing that doctors are always present when irradiating ranges from 08h30 to 17h00, which means that $\underline w^{t} = 60$, $\overline w^{t} = 570$, $\forall t \in \mathcal {T}$. A total of 12 care plans (21% of the patient population) will require patients to be scheduled between 08h30 and 17h00. In the NKI, patients belonging to a total of 4 care plans (namely stereotactic and hypofractionation schemes) need at least 2 days (48h) break between two sessions, corresponding to a total of 6% of the patient population. Moreover, in the NKI a maximum of six new patients are allowed to start treatment on the same linac and same day, thus *C* = 6.

In the NKI, patient preferences, i.e. the preferences given by patients for the desired starting time of their irradiation sessions, are taken into consideration when scheduling irradiation sessions. However, data regarding patient preferences are not currently recorded in the clinic, and thus real data for patient preferences cannot be used. Interviews with the appointment planners showed, according to their empirical knowledge, that around 1 in 4 patients will have a preference for the early morning (< 09h00), and 1 in 4 patients will want to receive their sessions in the end of the day (> 16h00). From there, we assumed that all other patients, including those who do not have a preference, will be included in the group of the remaining 50% (09h00–16h00). Therefore, we randomly generated time window preferences $[t_{i}^{\min \limits },t_{i}^{\max \limits }]$ for both urgent and regular patients as follows: 25% of patients have a preference for the morning window (*w*_1_ = [0,90]), 25% have a preference for receiving their RT sessions later in the day (*w*_3_ = [510,600]), while the remainder 50% of patients have a preference set for the time in between (*w*_2_ = [90,510]). A sensitivity analysis on the possible variations on these proportions is presented in Section 4.4.

### Results for several instance sizes

To test our model, we generated a set of test instances with various sizes regarding the number of patients ($|\mathcal {P}|$) and available linacs ($|\mathcal {K}|$) for a planning horizon of one labour week ($|\mathcal {T}| = 5$). We generated patient data by using historical data from the RT department of the NKI, a large cancer center operating in the Netherlands provided with 8 full-time working linacs and scheduling an average of 260 patients per week.

We started by running experiments using the MILP model alone. In each test instance, the patient-to-linac ratio of the NKI, i.e. 33 patients per linac per week, is maintained. Given that we are scaling down the NKI problem for a subset of the linacs, the pre-allocation of linacs to patient groups in Appendix A cannot be applied. Thus, we consider that all patients can be treated by all linacs ($\mathcal {K}^{i} = \mathcal {K}, \forall i \in \mathcal {P}$) by relaxing constraints (9).

As we can observe in Table 2, the proposed MILP model is able to find an optimal solution that schedules all sessions within the desired time window for all instance sizes up to 66 patients and 2 linacs within the CPU time limit. For the instance with 2 linacs, the proposed formulation proved effective in finding the optimal weekly schedule in less than 10 min of CPU time. However, the CPU time limit of 8 h was achieved for instances with 99 patients and higher, possibly due to complexity introduced by the exponentially higher number of variables and constraints. Since the model was able to solve the problem for one linac in just 100 s, we used Algorithm 1 to pre-allocate patients to linacs as a pre-processing step (considering that all linacs are empty, i.e. $WL(k)=0,\forall k \in \mathcal {K}$). We then apply the MILP model to solve the problem for each linac independently. We denote this combined approach as “heur+milp” in Table 2. We found that the combined approach is able to find a near-optimal solution in a total CPU time of 55 min or less for all instance sizes. For the NKI size (260 patients and 8 linacs), as few as 31 in 925 sessions were scheduled outside the desired window in just 7 min of CPU time. The solutions obtained by the combined approach schedule at most 6.2% of the sessions outside the preferred time window, with the percentage lowering down to 2% for the instance where the optimal solution is known (2 linacs). This indicates that the solutions found by partitioning the problem are probably even closer to optimality than the obtained percentages. Moreover, sessions scheduled outside the time window were, on average, under 30 min away from the corresponding windows. This shows that, with our approach, even sessions that are scheduled outside the preferred time window are still close to the target window bounds.

**Table 2:** Results of the MILP model for several instance sizes using NKI patient data

# patients	# linacs	# sessions scheduled		# sessions outside window		average deviation (min)		CPU time (s)	
		milp	heur+milp	milp	heur+milp	milp	heur+milp	milp	heur+milp
33	1	123	123	4	4 (3.25%)	5	5.0	99.9	99.9
66	2	254	247	1	5 (2.0%)	15	8.0	499.6	17.2
99	3	–	379	–	23 (6.1%)	*	16.5	*	218.7
132	4	–	361	–	22 (5.2%)	*	28.2	*	1780.5
165	5	–	580	–	33 (5.7%)	*	24.8	*	1175.8
198	6	–	700	–	40 (5.7%)	*	24.0	*	3318.3
231	7	–	810	–	50 (6.2%)	*	25.3	*	2705.2
260	8	–	925	–	31 (3.4%)	*	13.2	*	393.5

### Results for the NKI size

In this section, we combine Algorithm 1 with the MILP model to solve the problem for the NKI size but now including the pre-allocation constraints (9). This means that patients undergoing treatment are pre-assigned to the linac they have been receiving their treatment, which is assigned randomly based on historical data. Therefore, the initial workload values *WL*(*k*) of each linac *k* are pre-processed before running the cycle of Algorithm 1 to allocate new patients to linacs. Table 3 shows the results obtained after running Algorithm 1 for the 260 patients generated for the NKI size instance. As we can see, the pre-assignment solution provides a balanced workload amongst linacs, with an average of 2051.3 min and a maximum workload difference of 80 min between any pair of machines. Moreover, the number of patients assigned to each linac is considered stable, with a standard deviation of 3.3 patients amongst the 8 linacs at an (expected) average of 33 patients per linac. Utilization rates representing the percentage of the daily capacity (3000 min) assigned to each linac show that linacs have an utilization rate that ranges between 68.0% and 69.5%. This confirms the availability of slack capacity to apply the proposed heuristic effectively. The available capacity can be used to accommodate urgent patients arriving and having to start treatment during the planning horizon.

**Table 3:** Pre-assignment results after running Algorithm 1 for the NKI test instance

Linac	No. patients	Workload (WL)	Utilization
	assigned		rate (%)
L1	35	2055	68.5%
L2	27	2050	68.3%
L3	33	2055	68.5%
L4	39	2085	69.5%
L5	32	2040	68.0%
L6	32	2005	66.8%
L7	32	2080	69.3%
L8	30	2040	68.0%
avg	32.5	2051.3	68.4%
st. dev.	3.3	23.4	0.8%

Following the pre-assignment of patients to linacs, we cluster the linacs (L1, ... , L8) in different groups based on the characteristics of the available machines at the NKI. Thus, new patients can still be assigned any linac belonging to the cluster his/her linac belongs to. For instance, if a cluster contains L1 and L2, a new patient pre-assigned to L1 can still be assigned to L2 by the MILP model (if that patient type can be treated in L2). Moreover, from the 8 NKI linacs running on a daily basis, two (L7 and L8) are located in a satellite location. Decisions on whether patients will be receiving treatment in the satellite or the main location are made right after referral. In those cases, the pre-assignment cannot be changed between the main and satellite locations by the moment our model is intended to be used, which is at the beginning of the planning horizon. This means that patients pre-assigned to L7 can still be allocated to L8 and vice-versa, however the linac assignment cannot be changed by any of the remaining 6 linacs running on the main location. Furthermore, we have clustered the linacs by level of similarity in terms of the total patient volume that they are able to treat in common according to Appendix A, while ensuring that L7 and L8 are not clustered together with other linacs. Each experiment runs the MILP model sequentially for a certain number of times, which corresponds to the number of clusters. Table 4 outlines the experimental setup and its considered clusters, as well as the corresponding results in terms of solution quality and computational (CPU) time. In the first experiment, each linac is a cluster in and of itself and therefore the MILP model is run 8 times in a row. For this test instance, a cumulative deviation of 1085 min over a total of 41 sessions scheduled outside the desired window (out of 918) was achieved, in a combined CPU time of around 5 h. This means that, in less than 5 min of running time, our method was able to find a feasible solution with only 4.5% of the sessions being scheduled outside the preference time window. When 4 clusters are used, results are improved further. The extra flexibility provided by having 2 linacs per cluster decreased the overall objective value to 545, with as few as 26 sessions out of 936 (2.8%) being scheduled outside the intended time window. The combined CPU time was also reduced to around 3.5 h. The CPU time limit of 8 h per run was achieved when applying the same methodology for 2 clusters (main and satellite locations). Nevertheless, the solver was able to find a feasible solution, which did not improve the solution found with 4 clusters. A higher number of sessions (60) were found breaching the time window preference, in a total of 928 sessions scheduled. Note that, with the introduction of constraints (9), the MILP model was able to find a feasible solution the problem for an instance size of 6 linacs (L1, ... , L6 in the experiment with 2 clusters) during the computation time, although the CPU time was achieved in one of the runs. As with the experiments conducted in Section 4.3, the 8 h of CPU time limit was achieved when attempting to solve the problem for the 8 linacs combined in a single cluster without any integer solution being found. We also note that, overall, the performance of the combined approach decreases in terms of CPU time (from 7 min to 5 h) after considering the pre-allocation constraints (9) as part of the pre-assignment process. This shows that the existence of such pre-allocation, such as the one in the NKI, may decrease the performance of the proposed methodology by providing less flexibility when pre-assigning patients to linacs.

**Table 4:** Results for pre-assignment heuristic and MILP model for the NKI size

No.	Linacs belonging to each cluster	Cumulative	# sessions	# sessions	Average	Total
clusters		deviation	scheduled	outside	deviation	CPU time
		(obj. value)		window	(min)	(s)
8	[L1] [L2] [L3] [L4] [L5] [L6] [L7] [L8]	1085	918	41 (4.5%)	26.5	18143
4	[L1,L3] [L2,L6] [L4,L5] [L7,L8]	545	936	26 (2.8%)	21.0	11763
2	[L1,L2,L3,L4,L5,L6] [L7,L8]	7695^[^a]	928^[^a]	60 (6.5%)^[^a]	128.3^[^a]	31181^[^a]
1	[L1,L2,L3,L4,L5,L6,L7,L8]	–	–	–	–	–

^a^ CPU time limit achieved in at least one cluster

### Sensitivity analysis

In this part, we investigated the impact of varying two input parameters: the probability breakdown for the possible time windows being chosen by patients, and the size (minutes) of the time windows being made available to patients. In our sensitivity analysis, we vary the size of both time windows *w*_1_ and *w*_3_ from 90 min to 120 and 150 min, and test the probability breakdown [*w*_1_,*w*_2_,*w*_3_] at [50%,25%,25%] and [12.5%,75%,12.5%] in addition to the original [25%, 50%, 25%]. All combinations between these scenarios are tested using the instance with 4 clusters, since it has shown to provide the best performance regarding both the solution quality and overall CPU time needed (Table 4). The maximum allowed CPU time in these experiments is set to 7200 s (2 h) per run, for a combined CPU time limit of 28800 s (8 h). Results (Tables 5, 6, 7 and 8) show that the patient satisfaction levels may significantly increase by enlarging the window size to 120 min, with the percentage of sessions being scheduled outside the preferential window decreasing to 0.5% (baseline). Besides, the total CPU time needed to solve the 4 subproblems associated with the original problem decreases from 3.5 h (11299 s) to only 108 min. On the other hand, extending the windows *w*_1_ and *w*_3_ to [0,150] and [450,600], respectively, allowed our methodology to schedule all sessions within the desired time window in just 43.3 s when the baseline probabilities are maintained.

**Table 5:** Total cumulative deviation, in minutes

Total deviation (Obj. value)			
Probabilities (*w*_1_ / *w*_2_ / *w*_3_)	Window size (min)		
	150	120	90
0.25 / 0.5 / 0.25	0	65	650
0.5 / 0.25 / 0.25	320	4659^[^a]	25614^[^a]
0.125 / 0.75 / 0.125	0	0	55

^a^ CPU time limit achieved

**Table 6:** Percentage of sessions breaching the time window preference

% sessions outside window			
Probabilities (*w*_1_ / *w*_2_ / *w*_3_)	Window size (min)		
	150	120	90
0.25 / 0.50 / 0.25	0.0%	0.5%	3.4%
0.5 / 0.25 / 0.25	1.5%	10.1%^[^a]	28.8%^[^a]
0.125 / 0.75 / 0.125	0.0%	0.0%	0.5%

^a^ CPU time limit achieved

**Table 7:** Average deviation, in minutes, of the sessions outside window

Average deviation (min)			
Probabilities (*w*_1_ / *w*_2_ / *w*_3_)	Window size (min)		
	150	120	90
0.25 / 0.50 / 0.25	0.0	13.0	20.3
0.5 / 0.25 / 0.25	22.9	50.1^[^a]	97.4^[^a]
0.125 / 0.75 / 0.125	0.0	0.0	11.0

^a^ CPU time limit achieved

**Table 8:** CPU time, in seconds, of the 4 runs combined

Total CPU time (s)			
Probabilities (*w*_1_ / *w*_2_ / *w*_3_)	Window size (min)		
	150	120	90
0.25 / 0.50 / 0.25	43.3	108	11299
0.5 / 0.25 / 0.25	5341.5	18981^[^a]	28800^[^a]
0.125 / 0.75 / 0.125	60.8	42	39

^a^ CPU time limit achieved

By changing the probability of patients choosing *w*_1_ from 25% to 50%, we observe that the complexity of the problem increases substantially. In fact, with the increased competition level for a window size of 90 min, the CPU time limit of 7200 s was reached in all 4 clusters. For a window size of 120, 2 out of the 4 runs achieved the time limit before optimality could be proved. Still, a feasible solution has been found in both cases, with only 10% of the sessions being booked outside the desired window for the 120-min case, and approximately 29% time preference breaching rate when the window size is equal to 90 min. An opposite phenomena is observed when the probabilities are set to [12.5%, 75%, 12.5%], with an ideal solution (all sessions within the desired time window) being found for the 150 and 120-min time window sizes, and only 0.5% of sessions being scheduled outside the time window for the 90-min case. All the instances for this last probability breakdown were solved in under a minute of total CPU time.

## Discussion

The proposed MILP model has proven to be efficient in achieving an optimal solution for small instances of up to 66 patients and 2 linacs. For larger cancer centers (3 linacs or more), the combination of the pre-assignment heuristic and the MILP model was able to provide near-optimal solutions (maximum of 6.2% optimality gap) quickly (less than 1 h), thus ensuring the scalability of our combined approach. Given the elevated fulfillment rates provided by these solutions, RT centers may opt for the combined approach in order to ensure low computation times without loosing significant levels of solution quality. By running the combined approach for the NKI size considering pre-allocation constraints and patients with ongoing treatments, the combination between the MILP model and the heuristic procedure allowed to find a solution in which as few as 2.8% of the sessions are scheduled outside the preference time window in around 3.5 h of CPU time. The positive performance of our methodology when partitioning the problem suggests that large RT centers should divide the main problem in subproblems (subsets of linacs) and use the MILP model to solve each subproblem separately. Centers may split the fleet of linacs based on their location, technological specifications (e.g. cone-beam CT embedded or not) or based on staff planning. Although the obtained solution for the original problem may not be proven optimal, we believe that our methodology can be effective in generating a (near-)optimal schedule in due time for most real-world RT centers. According to the International Atomic Energy Agency (IAEA), the NKI ranks amongst the largest RT centers in the Netherlands with a total of 10 radiation machines (orthovoltage machines included) registered by 2017 [19], just below the Erasmus University Medical Center (12) and the University Medical Centre Utrecht (11) but above all the other 21 Dutch centers. Comparing with the US, the NKI size matches those of the largest cancer centers, paired with e.g. the Stanford Hospital (11) and the New York Memorial Sloan-Kettering Cancer (10).

A sensitivity analysis revealed that the larger the preferential time window, the easier it is for our approach to fit the irradiation sessions within the corresponding window preferences. Although enlarging the window can be seen as an advantage from a model viewpoint, we lack evidence on whether the patient would still be satisfied with such window size. Moreover, it has been verified that when the competition for the same time window increases (from 25% to 50% of the patients), the size of the time window to be chosen must be enlarged (from 90 to 150 min) in order to keep the computation times low. RT centers using the proposed approach should then monitor the percentage of patients asking for their sessions for the same period, and re-dimension the time window put available accordingly.

While we believe our model captures the operational constraints encountered in the vast majority of RT centers, there may be need to consider additional features in the model for a practical implementation. For instance, it is known that most patients need to have a weekly consultation with the radiation oncologist in charge of the follow-up during the course of their treatment. A possible extension of the model could be to include the availability of doctors’ agenda to ensure a proper coordination between the weekly consultation and (one of the) treatment sessions. Moreover, linacs need to undergo maintenance on a certain frequency basis. Maintenance operations are usually undertaken during office hours, with the linac under maintenance being replaced by a “back-up” linac. In the NKI, this linac is not able to treat certain care plans, since it does not have an embedded cone-beam CT scanner. Therefore, an operational offline re-scheduling of patients with a proper allocation to other machines may be needed when implementing the solution output by our model.

In case some restrictions (e.g. constraints (12) or (15)) are not part of the planning process of the RT center interested in using our methodology, the MILP model can be easily manipulated to account for those differences by relaxing (excluding) or adding constraints before solving the problem. Furthermore, the solution found by our model can be merely used as a basis where adaptations that fit specific needs of individual patients can be integrated to build a more robust, personalized solution. On the other hand, collection of real data and information regarding patient preferences would further increase the robustness of our solutions. Although planners and clinicians have provided insights on the usual requests asked by patients based on their experience, real data on patient perspectives regarding the desired time window sizes and actual time preferences for appointment times would allow for more concrete and realistic conclusions. For instance, some patients may be more interested in consistency amongst the appointment times of their treatment sessions rather than specific time preferences for their appointments. Other patients may not have a preference at all, being more interested in starting treatment as soon as possible. In these cases, one may use the extra flexibility associated with those patients to further improve the fulfilment of requests of patients who actually have a preference. Moreover, in case some RT centers do not consider patient preferences when scheduling irradiation sessions, they may still use our approach to ensure consistency between appointment times. For instance, a “fictitious” time window of a pre-defined size may be chosen for each patient before running the MILP model, which then outputs a solution with the desired degree of consistency amongst appointment times for each patient.

A possible extension of our approach could be to apply a formulation inspired in the Multi-Mode Resource Constrained Project Scheduling Problem (MMRCPSP) [20]. For example, the linacs could be modelled as a renewable resource with a fixed capacity, and variables *X* decide upon a “mode”, defined as the combinations of linacs and time slots. Time window violations can be calculated using the finish-start precedence relations between activities, computed as time increments [20].

Efforts to assess the practical feasibility of the obtained solutions need to be performed by RT managers and/or planners before using the model in practice. This and other implementation steps are currently being made together with department managers and clinicians of the NKI in order to perform an implementation of the proposed methodology in the clinic.

## Conclusions

Earlier research on the problem of scheduling RT sessions considering time windows is scarce, with existing models being developed in the context of particle therapy, which makes them directly non-applicable to conventional RT. In this study, we propose a MILP model that is able to solve the RT scheduling problem to optimality in reasonable computation time for RT centers with up to 2 linacs. For RT centers with 3 linacs or more, we propose a heuristic procedure that is capable of pre-assigning patients to linacs while maintaining a balanced workload between linacs. Combining the pre-assignment heuristic with the MILP model allowed to solve the problem in less than 3.5 h of CPU time with 97.2% of the sessions scheduled within the desired time window for a large cancer center operating with 8 linacs.

Besides providing automated decision making for scheduling RT treatments which allows managers and planners of RT centers to save time and effort during the scheduling process, our algorithm is capable of incorporating patient preferences while ensuring that all timeliness, medical and technical constraints are take into account. Since the modeled problem and corresponding assumptions are standard among RT centers and the patient mix at the NKI is representative of the patient population found in RT in general, our methodology can be generally applied to RT centers.

## Appendix

**Table 9:** Pre-allocationof care plans to linacs in the RT department of the NKI (2017)

ID	Careplan	# patients	L1	L2	L3	L4	L5	L6	L7	L8
1	Anus +/- inguinal lymph node	22	X	X	X	X	X	X	X	X
2	Adrenal stereotaxic	4	X	X		X				
3	Bladder	43		X						X
4	Bladder (partial)	22		X						X
5	Chest wall	45	X	X	X	X	X	X	X	X
6	Chest wall+axilla	114	X	X	X	X	X	X	X	X
7	Chest wall+axilla+parasternal	15	X							
8	Chest wall+parasternal	3	X							
9	Chest wall (bsu)	2							X	
10	Bone metastasis	1099	X	X	X	X	X	X		
11	Bone metastasis stereotaxic	52	X	X		X				X
12	Uterus	42		X		X				
13	Endometrium	16	X	X		X				X
14	Neck (bsu)	9	X		X	X	X	X	X	X
15	Brain 1 fraction	107		X					X	X
16	Brain several fractions	86		X	X				X	X
17	Brain (whole)	159	X	X	X	X	X	X	X	X
18	Brain electrons	6	X							
19	Lymph node stereotaxic	17	X	X		X				X
20	Head-and-neck	230	X		X	X	X	X		
21	Head-and-neck (palliative)	4	X		X	X	X	X		
22	Larynx 2vs	2		X						
23	Liver	6	X	X		X				
24	Lung (palliative)	61	X	X	X	X	X	X	X	X
25	Lung	273	X	X	X	X	X	X	X	X
26	Lung (bsu)	47	X	X	X	X	X	X	X	X
27	Lung stereotaxic	206	X	X		X	X	X		X
28	Lymphoma	44	X	X	X	X	X	X	X	X
29	Lymphoma (bsu)	19	X	X	X	X	X	X	X	X
30	Stomach	14	X	X	X	X	X	X	X	X
31	Breast	777	X	X	X	X	X	X	X	X
32	Breast+axilla	179	X	X	X	X	X	X	X	X
33	Breast+axilla+parasternal	20	X	X	X	X	X	X	X	X
34	Breast+parasternal	1	X	X	X	X	X	X	X	X
35	Esophagus	77	X	X	X	X	X	X	X	X
36	Esophagus (palliative)	22	X	X	X	X	X	X	X	X
37	Axilla (virtual)	5	X	X	X	X	X	X	X	X
38	Orbit (eye socket)	1		X						X
39	Ovaries	5	X	X		X				X
40	Others	183	X	X	X	X	X	X	X	X
41	Others (bsu)	60	X	X	X	X	X	X	X	X
42	PAO (+/- iliac single-sided)	1	X	X		X				X
43	Penis	11	X	X		X				X
44	Prostate	243	X	X	X	X	X	X	X	X
45	Prostate+pelvic lymph nodes	60	X	X	X	X	X	X	X	X
46	Prostatic bed	36	X	X	X	X	X	X	X	X
47	Prostatic bed+pelvic lymph nodes	25	X	X	X	X	X	X	X	X
48	Rectum / sigmoid	87	X	X	X	X	X	X	X	X
49	Rectum 13 x 3 Gy	5	X	X	X	X	X	X	X	X
50	Rectum 5 x 5 Gy	56			X		X		X	
51	Sarcoma abdominal/thoracic wall	13	X		X	X	X	X	X	X
52	Sarcoma extremity	28	X		X	X	X	X	X	
53	Sarcoma retroperitoneal	9	X		X	X	X	X	X	
54	Spinal cord	33		X						
55	Vagina	5	X	X		X				X
56	Vulva +/- inguinal lymph nodes	9	X	X	X	X	X	X	X	X
Total		4720								
